# Plant-Associated Microbes: From Rhizobia To Plant Microbiomes

**DOI:** 10.1264/jsme2.ME3301rh

**Published:** 2018-03-29

**Authors:** Sawa Wasai, Kiwamu Minamisawa

**Affiliations:** 1 Graduate School of Life Sciences, Tohoku University Katahira, 2–1–1 Aoba-ku, Sendai, Miyagi 980–8577 Japan

Plant environments provide a diversity of ecological niches for microorganisms including rhizobia, plant growth-promoting microbes, and pathogens. Among them, rhizobia have been extensively studied for their dynamically-changing genome structures, polyphasic interactions with host plants, and biogeochemical functions as representative plant-associated microbes. Here, rhizobia are collectively termed as nodule-forming N_2_-fixing bacteria, including the genera *Rhizobium*, *Bradyrhizobium*, *Mesorhizobium*, *Ensifer* (*Sinorhizobium*), and *Azorhizobium*.

Rhizobial genes for nodulation (*nod*) and nitrogen fixation (*nif*) appear to be acquired by genomes using lateral gene transfer. Recent studies provided several lines of evidence for the horizontal transfer of symbiosis islands, which is a type of adaptation process to host legumes from soil bacteria (5, 21, 46). Kasai-Maita *et al.* (15) demonstrated the dynamics of symbiosis islands in three strains of *Mesorhizobium loti*: the integration of symbiosis islands into a phenylalanine-tRNA gene and subsequent genome rearrangement. Evidence for the horizontal transfer of symbiotic genes was also found in the phylogenetic relationships of the *nodC* and 16S rRNA genes of hairy vetch rhizobia (54) and by the presence of identical *nodD* and *nifD* sequences in the *Bradyrhizobium* and *Ensifer* species of Afghanistan isolates from soybean nodules (4). Thus, it is widely accepted that the horizontal transfer of symbiosis islands and genes frequently occurred between rhizobia and other soil bacteria.

*Bradyrhizobium* sp. DOA9, a non-photosynthetic bacterial strain originally isolated from the root nodules of *Aeschynomene americana*, efficiently nodulates on the roots of many leguminous plants. The genome is composed of a single chromosome and single megaplasmid (pDOA9) with symbiotic genes (31, 49), which is less common than the genome structures of many other symbiotic bradyrhizobia (13, 14). Okubo *et al.* (34) compared the *nifDK* gene sequences of rhizobial and non-rhizobial *Bradyrhizobium* strains in order to examine the evolutionary history of *nif* genes in the genus *Bradyrhizobium*, and suggested that the *nif* genes on symbiosis islands were forced to reduce GC contents with higher substitution rates than the ancestral sequences. On the other hand, the *nifDK* genes on the megaplasmid pDOA9 were derived from the non-symbiotic loci of *Bradyrhizobium* with similar evolutionary rates to the ancestral sequences. The low GC pressure in *nif* genes on symbiosis islands may be related to the evolutionary processes of symbiotic *bradyrhizobia* through associations with plants.

Whole-genome sequencing and post-genomic studies on rhizobia have facilitated our understanding of their lifestyle and strategies to adapt to environmental conditions. The symbiotic systems are regulated by many environmental cues, such as legume host flavonoids (47), plant hormone regulators (50), temperature (43), CO_2_ concentrations (45), and rhizobial systems, including sigma factor (27) and cell division and differentiation (9, 29). The distribution patterns of bradyrhizobial species and genotypes appear to be associated with geographic locations (43) and soil types (42) in Japan, which are more likely explained by the capabilities of anaerobic nitrate respiration (38, 44) and uptake hydrogenase (24).

Recent investigations have focused on the interactions between non-rhizobial bacteria and plants. For example, the inoculation of specific bacteria into plant seedlings has been shown to promote the growth of a number of plants, such as potato, rice, and cacao. Tchinda *et al.* (48) isolated many *Actinobacteria* stains from cacao pods, and evaluated the promotion of plant growth with their siderophore production and biosynthesis of 1-aminocyclopropane-1-carboxylate (ACC) deaminase and indole-3-acetic acid (IAA). These findings suggested that the *Actinobacteria* strains colonizing cacao pods function as plant health agents. A similar approach was conducted for *Novosphingobium* strains to optimize rice cultivation (36). N_2_-fixing *Novosphingobium* strains were isolated from rice plant tissue and their effects on the promotion of plant growth were tested under nitrogen-free conditions. The selected strains of *Novosphingobium* effectively colonized within rice plant interiors and consequently promoted its growth.

Not only the function of a single bacterial strain, but also the synergetic functions of different bacterial species for the promotion of plant growth have been studied (22, 39). Bacterial strains from potato roots and tubers were initially tested in order to establish whether they produced plant growth-promoting substances or had positive or negative effects on plant growth (39). The co-inoculation of two different bacterial species exerted stronger effects on plant growth than the inoculation of any single species, suggesting that the synergetic functions of multiple strains were more effective on plant-bacteria interactions than those of a single specific strain (39).

Although plant-pathogenic bacteria cause significant damage to agricultural production, some endophytic bacteria protect against pathogenic infections and subsequent disease expression. Hassan *et al.* (6) clearly showed that the endophytic colonization of *Streptomyces humidus* MBCN152-1 in cabbage plug seedlings increased host plant weight and protected against disease expression caused by *Alternaria brassicicola*, with the percentage of diseased seedlings becoming less than 10% with, but approximately 40% without the inoculation of strain MBCN152-1. Hieno *et al.* (7) investigated the molecular mechanisms of action of endophytic bacteria against the possible pathogen *Pseudomonas syringae* pv. *tomato* DC3000 (Pst) in *Arabidopsis thaliana*. They demonstrated that the *MYB44* gene of *Penicillium*, a transcription factor and stomata-specific enhancer of the ABA signal for the stomatal closure of *Arabidopsis thaliana*, appeared to function by preventing the penetration of pathogens through stomata, which is one of the mechanisms protecting against plant diseases.

Recent studies on plant-associated bacteria have been depending more significantly on culture-independent omic analyses of microbial ecologies than on conventional cultivation-based techniques (8, 12, 28). Consequently, plants and their microbiota may be regarded as holobionts, which embrace multiple plant-microbe and microbe-microbe interactions (3, 37, 53).

Rice is one of the most important cereal crops in the world and is grown mainly in flooded paddy fields. Important biogeochemical processes including the emission of methane, a greenhouse gas, occur actively in paddy rice environments, and the rhizosphere in a paddy field is considered to be a hot spot for the various inorganic redox reactions of carbon, sulfur, and nitrogen compounds (17). Thus, the microbiomes of paddy rice play an important role in carbon, sulfur, and nitrogen biogeochemical cycles (17). An early metagenome analysis indicated that rice shoot microbiomes were dominated by members of *Alphaproteobacteria* (51–52%), *Actinobacteria* (11–15%), *Gammaproteobacteria* (9–10%), and *Betaproteobacteria* (4–10%) (32). Members of the uncharacterized phylum *Planctomycetes* were also abundant in leaf sheaths (11). Shoot microbiomes harbored more abundant genes for C1 compound metabolism and ACC deaminase than the rhizosphere microbiome (32). In contrast, the root microbiomes of paddy rice were significantly influenced by different environmental conditions, such as nitrogen fertilizer amendments (10, 41), atmospheric CO_2_ concentrations (33), rice growth stages (33), temperature (33), and rice genotypes (26). New findings have been obtained from these metagenomic studies. A rice symbiotic gene (*OsCCaMK*), relevant to rhizobial nodulation and mycorrhization in plants, appeared to function in the accommodation of N_2_-fixing methanotrophs in root tissues under low-N fertilizer management conditions, which may lead to nitrogen utilization by host plants via bacterial N_2_ fixation (26). Thus, CH_4_ oxidation and methanotrophs are considered to be a driving force for shaping bacterial communities in rice roots in CH_4_-rich environments (26). Amplicon sequence analyses of the 16S rRNA gene indicated that rice root microbiomes responded to *Azospirillum* sp. B501 inoculations (2) and sulfur amendments (23). The abundance of uncharacterized phylum ™7 members in rice roots was increased by sulfur amendments (23). In addition, an inoculation experiment of non-photosynthetic *Bradyrhizobium* sp. strain SUTN9-2 indicated that the type III Secretion System (T3SS) of the bacterium is one of the key mechanisms for endophyte colonization in rice roots (35).

Root microbiomes have also been characterized by 16S rRNA gene sequencing in other plants, including sugar beet (30, 52), *Arabidopsis* grown under different conditions of nitrogen availability (18), potato genotypes resistant and susceptible to *S. turgidiscabies*-induced disease (16), and the healthy garden plant, *Anthurium andraeanum* (40). In *A. andraeanum*, the different tissues of the leaf, stem, root, spathe, and spadix had often specific microbiomes (40). By amplicon sequencing of the 16S rRNA gene, Lee *et al.* (19, 20) compared the microbial community composition in soil in which tomato plants were planted with and without *Ralstonia solanacearum* wilt symptoms, and suggested that several genera of components (*e.g.*, *Hephaestia*, *Azospirillum*, *Dyella*, and *Choloroflexi*) may contribute to suppressing the soil-borne pathogens of bacterial wilt. In a metagenome analysis, Minami *et al.* (25) found that *Methylobacterium* species dominated in the shoot microbiomes in soybean plants. A functional gene analysis also indicated the abundant occurrence of genes for urea degradation, such as the urease of *Methylobacterium* species (25). This study demonstrated that ureide may serve as an important nitrogen source of shoot-associated microbes even though it is a key substance of fixed nitrogen transportation from legume nodules to shoots.

We would like to introduce some of the future perspectives in plant microbiome research to further address community-level functions. New experimental approaches have recently been developed for plant microbiome research: synthetic engineering approaches to plant microbial communities in gnotobiotic systems (1, 53) and informatics approaches to the identification of “hub microbes” (51, 53). The integration of these new approaches with conventional techniques and knowledge as described herein will open a new dimension of plant microbiomes and their application to agriculture.
